# rAAV2-Mediated Restoration of GALC in Neural Stem Cells from Krabbe Patient-Derived iPSCs

**DOI:** 10.3390/ph16040624

**Published:** 2023-04-20

**Authors:** Guoshuai Tian, Chunyu Cao, Shuyue Li, Wei Wang, Ye Zhang, Yafeng Lv

**Affiliations:** 1 State Key Laboratory of Medical Molecular Biology, Department of Biochemistry and Molecular Biology, Institute of Basic Medical Sciences Chinese Academy of Medical Sciences, School of Basic Medicine Peking Union Medical College, Beijing 100005, China; tgs@spaces.ac.cn; 2 Hubei Key Laboratory of Tumor Microenvironment and Immunotherapy, College of Basic Medical Sciences, China Three Gorges University, Yichang 443000, China; 3 Department of Neurology, China-Japan Friendship Hospital, Beijing 100029, China

**Keywords:** Krabbe disease, NSCs, rAAV vector, gene therapy, GALC, psychosine

## Abstract

Krabbe disease is a rare neurodegenerative fatal disease. It is caused by deficiency of the lysosomal enzyme galactocerebrosidase (GALC), which results in progressive accumulation of galactolipid substrates in myelin-forming cells. However, there is still a lack of appropriate neural models and effective approaches for Krabbe disease. We generated induced pluripotent stem cells (iPSCs) from a Krabbe patient previously. Here, Krabbe patient-derived neural stem cells (K-NSCs) were induced from these iPSCs. By using nine kinds of recombinant adeno-associated virus (rAAV) vectors to infect K-NSCs, we found that the rAAV2 vector has high transduction efficiency for K-NSCs. Most importantly, rAAV2-GALC rescued GALC enzymatic activity in K-NSCs. Our findings not only establish a novel patient NSC model for Krabbe disease, but also firstly indicate the potential of rAAV2-mediated gene therapy for this devastating disease.

## 1. Introduction

Krabbe disease is a rare, autosomal recessive neurodegenerative lysosomal storage disease, which is also known as globoid cell leukodystrophy (GLD). This fatal disease results from the functional deficiency of galactocerebrosidase (GALC), a critical enzyme in lysosomes [1,2]. More than 130 GALC pathogenic variants have been documented, of which over 95% are correlated with Krabbe disease [3]. Pathogenic variants in GALC result in abnormal accumulation of the cytotoxic substrate, psychosine [4]. The myelin-forming oligodendrocytes in the central nervous system and Schwann cells in the peripheral nervous system are sensitive to the accumulated psychosine, which can cause the death of these important cell populations, leading to demyelination and neurodegeneration in the nervous systems [5].

From a clinical perspective, Krabbe disease could be divided into four subtypes based on the age of symptom onset: early infantile; late infantile; juvenile; adolescent, or adult [6]. Among those, the most serious one is the early infantile subtype (accounting for 85–90%) [7]. The patients are generally diagnosed before 6 months of age, and the common symptoms mainly include paralysis, stiffness, irritability, blindness, hearing loss, poor feeding, and seizures [8]. A number of infant patients will die from organ failure before 2 years of age if untreated, which is mainly caused by severe demyelination and neurodegeneration [9].

The only disease-improvement treatment, hematopoietic stem cell transplantation (HSCT), exhibits significant survival benefits for pre-symptomatic infants [10]. Nevertheless, after HSCT treatment, the motor performance of patients remains poor, and the peripheral neuropathy is still getting worse. It is presumably because Krabbe disease progressed rapidly, while the infiltration rate of transplanted cells to the nervous system is relatively slow [11]. Notably, only migrated to the nervous system can the engrafted hematopoietic stem cells perform therapeutic functions. For this reason, for those patients who already have symptoms, the treatment outcome of HSCT is normally unsatisfactory. Therefore, exploring novel treatment approaches for Krabbe disease is of certain medical importance.

Several animal models have been widely used to study pathological manifestations and test novel therapeutic approaches for Krabbe disease [12]. However, these models are considered not ideal. On one hand, most of them carry spontaneous mutations that are not found in humans. On the other hand, in general, they can only partially recapitulate the pathological events diagnosed in patients. Hence, although there have been some animal models, it still makes sense to build new Krabbe disease models. It has been reported that patient-derived neural cells with specific genetic backgrounds may be good neurodegenerative disease models and can be used to explore therapeutic approaches [13]. For Krabbe disease, so far as we know, there are still few patient-derived neural cell models and its-based curative gene delivery vector being reported [14,15].

In our previous study, we reported a 13-year-old male patient with two heterozygous pathogenic variants in GALC (c.461C > A, c.1244G > A) [16]. The patient’s clinical manifestations were peripheral neuropathy, with electro-physiological demyelinating characteristics and visual loss. Brain MRI images showed leukoencephalopathy. We also generated the first Krabbe disease-relevant induced pluripotent stem cells (K-iPSCs), and the fibroblast-derived K-iPSCs retained the GALC pathogenic variants [16]. iPSCs not only act as the platform to elucidate cellular and molecular defects but also can differentiate into various cell types, including neural stem cells (NSCs) [17,18,19]. NSCs are characterized by the capacity for self-renewal in the undifferentiated state and differentiation into glial and neuronal subtypes. NSCs could release several trophic factors into the microenvironment and restore neuronal cell populations [20,21]. Nevertheless, gene delivery to NSCs can regulate neurogenesis and thus, promote the development of regenerative medicine.

Recombinant adeno-associated virus (rAAV) is one of the most promising viral vectors for gene therapy. rAAV vectors are widely used for gene therapy, including several clinically approved products, such as Zolgensma and Upstaza [22,23]. Several rAAV serotypes have been reported thus far [24,25]. It is well known that rAAV serotypes can infect numerous cells, such as embryonic stem cells and fibroblasts [26,27]. However, few rAAV serotypes have achieved efficient infection of NSCs derived from human iPSCs [28]. In this study, we generated Krabbe patient-derived NSCs (K-NSCs) that were differentiated from K-iPSCs. K-NSCs were applied as an in vitro model to explore rAAV serotypes with high transduction efficiency. For the first time, we found that the rAAV2 serotype efficiently infected K-NSCs. Furthermore, gene delivery via the rAAV2 serotype was applied to rescue GALC enzymatic activity in K-NSCs. Our study provides evidence of the potential application of the rAAV2 serotype for gene therapy for Krabbe disease.

## 2. Results

### 2.1. K-NSCs Are Induced Successfully from K-iPSCs

**Figure 1:**
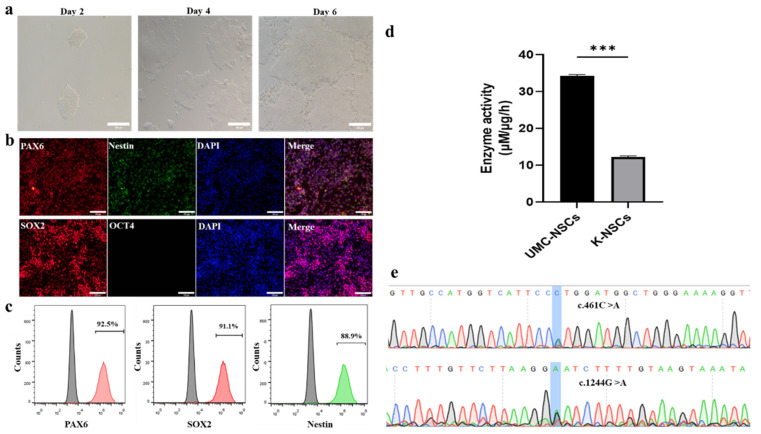
K-NSCs were differentiated from K-iPSCs. (**a**): Phase-contrast microscopy of K-NSCs during the induction process on day 2, day 4, and day 6. Scale bar, 100 μm. (**b**): Representative images of derived K-NSC (Day 6) immunofluorescence after staining with PAX6 (red), Nestin (green), SOX2 (red), and OCT4 (green) antibodies. Nuclei were stained with DAPI (blue). Scale bar, 100 μm. (**c**): NSC-specific protein expression was quantified by flow cytometry. Scale bar, 100 μm. (**d**): GALC enzymatic activity in K-NSCs and UMC-NSCs (Ctrl). Data are expressed as the mean ± SD (*n* = 4). *** *p* < 0.001. (**e**): Detection of the pathogenic variants (c.461C > A, c.1244G > A) of GALC in the K-NSCs via sanger sequencing.

To establish a human neural cell model of Krabbe disease, K-NSCs were induced from K-iPSCs. Figure 1a showed a phase-contrast picture of K-NSCs during the induction process. To assess whether K-NSCs were induced successfully, immunofluorescence staining was performed for NSC markers (Nestin, SOX2, and PAX6) and a pluripotency marker (OCT4). The results showed that K-NSCs were positive for Nestin, SOX2, and PAX6 but negative for OCT4 (Figure 1b). To quantify the induced efficiency, flow cytometry was performed and the results obtained for Nestin, SOX2, and PAX6 were consistent with the immunostaining data, which showed that approximately 90% of induced K-NSCs are capable of expressing those three NSC-specific proteins (Figure 1c). The GALC enzymatic activity of the K-NSCs was decreased versus that of control UMC-NSCs (NSCs derived from UMC-iPSCs, UMC-iPSCs were induced from the cord blood CD34+ cells) (Figure 1d). The pathogenic variants (c.461C > A, c.1244G > A) of GALC in the K-NSCs were detected via sanger sequencing. As shown in Figure 1e, the two heterozygous pathogenic variants retained in K-NSCs. The above results indicated that the K-NSCs might serve as a potential in vitro model of Krabbe disease. Therefore, K-NSCs were applied in subsequent research.

### 2.2. The rAAV2 Vector Efficiently Infects K-NSCs

**Figure 2:**
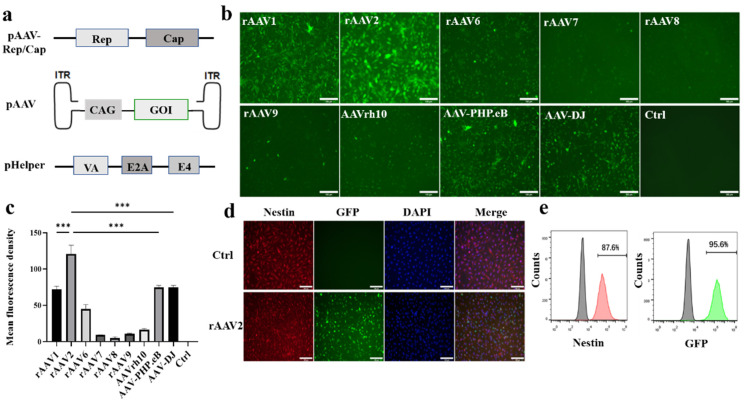
K-NSCs infected with rAAV remain undifferentiated. (**a**): Scheme of the three plasmids used for rAAV production. GOI, gene of interest, i.e., GFP or GALC. (**b**): GFP fluorescence in K-NSCs 48 h after infection with different GFP-encoding rAAVs at an MOI of 10^5^. Scale bar, 100 μm. (**c**): Mean fluorescence intensity of GFP in transfected K-NSCs quantified with ImageJ software (V1.8.0). Data are expressed as the mean ± SD (n = 5); *** *p* < 0.001. (**d**,**e**): The expression of GFP and Nestin in K-NSCs were evaluated 1-week post-infection by immunostaining and flow cytometry, respectively. Scale bar, 100 μm.

To find an rAAV serotype with high transduction efficiency for K-NSCs, nine kinds of rAAV serotypes with distinct capsids encoding green fluorescence protein (GFP), which generally applied in the pre-clinical research, were produced [23,29,30]. A schematic representation of the three plasmids used for rAAV generation is shown in Figure 2a. The pAAV-Rep/Cap and pHelper plasmids are necessary for rAAV packaging. A GFP-encoding sequence driven by a cytomegalovirus enhancer and chicken β-actin (CAG) promoter was inserted into the pAAV plasmid. Forty-eight hours post-infection with rAAV serotypes, the fluorescence intensity of GFP in K-NSCs was assessed by fluorescence microscopy. It was found that infection with the rAAV2-GFP vector resulted in abundant GFP expression at a multiplicity of infection (MOI) of 10^5^, which demonstrated that the rAAV2 serotype effectively transduced K-NSCs (Figure 2b,c). Furthermore, we evaluated whether the undifferentiated state of K-NSCs was altered seven days after rAAV2 infection. Immunofluorescence staining and flow cytometry for Nestin, an NSC marker, showed that K-NSCs maintained the expression of Nestin, indicating that rAAV2-infected K-NSCs remained undifferentiated 1-week post infection (Figure 2d,e).

### 2.3. rAAV2-GALC Restores GALC Enzymatic Activity in K-NSCs

**Figure 3:**
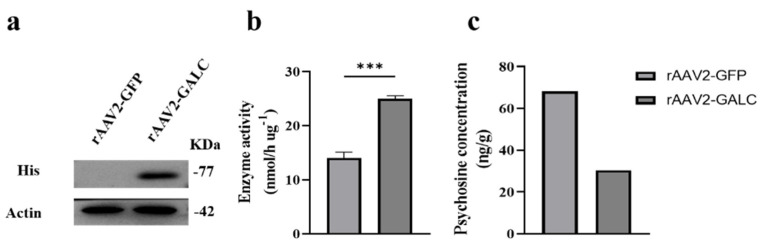
rAAV2-mediated GALC delivery to K-NSCs with high efficiency. (**a**): Western blot analysis of infected K-NSCs with antibodies against His (tagged with GALC) and actin. (**b**): rAAV2-GALC virus rescued the GALC enzyme activity of K-NSCs. Data are expressed as the mean ± SD (n = 4). *** *p* < 0.001. (**c**): rAAV2-GALC virus decreased accumulation of the GALC substrate psychosine in K-NSCs.

The above data showed that the rAAV2 serotype transduced K-NSCs efficiently and did not affect the undifferentiated state of K-NSCs. Nevertheless, it is crucial to assess the capacity of rAAV2 as a gene therapy vector in K-NSCs. Hence, rAAV2 encoding GALC tagged with the 6 x His peptide (rAAV2-GALC) was produced and used to infect K-NSCs. Notably, GALC was controlled by the CAG promoter. As a negative control, GFP was packaged into the rAAV2 serotype and delivered. The results showed that compared with rAAV2-GFP, rAAV2-GALC transduction resulted in an increased expression level of GALC and about a 2-fold restoration of GALC enzymatic activity (Figure 3a,b). As GALC hydrolyzes the lysosomal substrate psychosine, the psychosine concentration in K-NSCs infected with rAAV2-GALC and rAAV2-GFP was also measured. As shown in Figure 3c and Supplementary Figure S2, the abnormally increased psychosine levels decreased in rAAV2-GALC-treated K-NSCs. What is more, the neural progeny differentiated from K-NSCs maintained the expression of the GALC protein tagged with His (Supplementary Figure S3). These results illustrated that rAAV2 is a promising gene therapy vector for Krabbe disease.

## 3. Discussion

To date, treatment of Krabbe disease is still limited to HSCT. However, only when performed before disease onset could patients get survival benefits from HSCT. Notably, HSCT is not a curative approach, which could only delay the neurologic decline. Therefore, it is still necessary to explore novel treatments for Krabbe disease [10,31,32]. Due to the clear evidence that this disease is caused by the functional deficiency of GALC, gene therapy for GALC may be a direct and promising treatment approach for Krabbe disease. To achieve this goal, human-derived disease models for therapeutical research and effective gene delivery vectors are two crucial issues that need to be addressed. At present, several human cellular models for Krabbe disease have been established, including fibroblasts and hematopoietic cells [33,34]. Nevertheless, because Krabbe disease is a neurological disorder, previous cell models usually lack the capacity of recapitulating the molecular and cellular characteristics of neural cells [35]. Therefore, the establishment of neural model for this disease is of great importance.

NSCs serve as an excellent platform to study neurological diseases. NSCs can drive into a majority of neuronal cell types and are crucial for nervous system development, learning, and memory [25]. However, ethical restrictions make it unrealistic to obtain human NSCs directly. Thus, the pathogenesis and treatment of many neurological diseases, such as Krabbe disease, are difficult to explore [36]. Alternatively, the differentiation of iPSCs to NSCs is a feasible approach to obtaining the neurological model. Notably, iPSCs can be reprogrammed from human somatic cells obtained from the skin or the blood [37]. What is more, iPSCs provide a unique platform to explore the pathogenesis mechanism and potential curative strategies for some disorders, such as Alzheimer’s disease and Metachromatic Leukodystrophy [38,39,40]. As for Krabbe disease, to our knowledge, our laboratory established the first iPSCs model (K-iPSCs) and there are few reports concerning human-derived NSCs for Krabbe disease [15,16]. Here, we generated a novel NSC-based model in vitro by inducing K-iPSCs for the treatment of Krabbe disease.

Among the widely applied virus vectors, rAAV has emerged as an attractive vector for gene therapy, as it is nonpathogenic and infects a broad range of tissues and cells [41]. However, it was reported that the rAAV serotype infects stem cells with low efficiency [42,43,44]. Here, based on quantification by immunofluorescence staining and flow cytometry, we found that rAAV2 efficiently delivered the gene of interest to K-NSCs without affecting the undifferentiated state of the cells, which persisted for at least 1 week (Figure 2d,e). These results were consistent with reports from other researchers [15,43]. Previously reported NSCs that were transduced by the rAAV serotype with low efficiency were mainly derived from rodent or human embryos, indicating that species- and time-related heterogeneity may impact infection efficiency [42,43,44,45]. For example, human fetal NSCs were more easily infected by the rAAV2 serotype than human adult NSCs and murine NSCs [43]. There are numerous potential reasons for the low infectivity of rAAV in stem cells [26,27]. One of the widely accepted reasons is that some cells lack crucial receptors or coreceptors on the surface, such as heparan sulfate proteoglycan (HSPG), which plays a fundamental role in rAAV recognition and affinity [46]. The molecular mechanism of which rAAV2 has higher transduction efficiency to K-NSCs than other serotypes, found in this study, requires further investigation. Additionally, for expanding our finding to a broader Krabbe disease patient population, the transduction efficiency of rAAV2 needs to be further verified on more patient-derived NSCs with different pathogenic variants.

As a proof of concept, we have shown that the rAAV2-GALC vector restores GALC enzymatic activity and reduces the accumulation of the cytotoxic substrate psychosine in the K-NSCs (Figure 3), which supports prior literature that GALC addition could change the lipidomic profile [15]. It has been reported that GALC-deficient NSCs partially lose the ability to differentiate into various neural cell types, which is a presumable reason for neurodegeneration [47]. Thus, we assessed whether the rAAV2-transduced NSCs could generate GALC-expressed progeny. The results showed that GALC still expresses in the progeny of rAAV2-infected K-NSCs (Supplementary Figure S3). It is noteworthy that even though rAAV2 effectively infects K-NSCs and NSCs secrete specific trophic factors to restore neuronal cell populations, NSCs are still a single component of the disease model in vitro [20,21]. Compared with the three-dimensional brain of humans, NSCs lack complex structures and various cell compositions. To date, several studies applied cerebral organoids to recapitulate the development and pathological event of neural disorders [48,49]. It was found that the gene expression programs of cortical cells in organoids are remarkably similar to that of fetal cerebral cortex tissue, which indicates that the organoid containing multiple cell components could be used as another potential disease model to further explore rAAV2-mediated gene therapy for Krabbe disease [50].

Although rAAV2 efficiently transduces K-NSCs in vitro, it has difficulty crossing the blood–brain barrier (BBB) effectively, which limits the application with systematic administration [51,52]. Moreover, for Krabbe patients and murine or primate models, BBB is an unavoidable physiological structure to relieve clinical symptoms [53,54]. To increase the BBB crossing efficiency and infectivity of AAV, researchers have applied several routes of administration, including magnetic resonance imaging-guided focused ultrasound combined with microbubbles injected in the bloodstream (MRIgFUS) combined with intravenous injection, intra-parenchymal delivery, intra-cerebrospinal fluid delivery, and intrathecal delivery [55,56,57,58,59]. Of importance, a rAAV2-based product Upstaza has been approved for clinical application recently, whose route of administration is intraputaminal infusion [23]. Therefore, this approach may also be a potential administration route for gene therapy of Krabbe disease by rAAV2. Additionally, some studies achieved high performance to central nervous system using novel engineered rAAV variants, such as AAV-PHP.eB, AAV r3.45, and AAVrh10 [30,60,61]. Thus, engineering the capsid of the rAAV2 serotype to provide it with BBB crossing capacity may be also a feasible strategy. Overall, rAAV2-mediated in vivo gene therapy needs to be further explored, via optimization of the administration routes or engineering the capsid of rAAV2.

## 4. Materials and Methods

### 4.1. Antibodies

Antibodies for PAX6 (ab195045), OCT4 (ab184665), Nestin (ab18102), and His (ab18184) were purchased from Abcam (Cambridge, UK); Antibody for SOX2 (3579) was from Cell Signaling Technology (Danvers, MA, USA); Antibody for CAP (E-AB-13962) was from ARP (Danvers, MA, USA); Antibody for Actin (sc-8432) was from Santa Cruz Biotech (Santa Cruz, CA, USA).

### 4.2. NSC Induction and Culture

iPSCs derived from Krabbe patient (K-iPSCs, described as PUMCi001-A cells) [16], and UMC-iPSCs (a kind gift provided by Jie Na from Tsinghua University, induced from cord blood CD34+ cells) [62] were cultured on plates coated with Geltrex matrix (Gibco, MD) in Essential 8 medium (Gibco, MD) under feeder-free conditions with 5% CO_2_ at 37 °C. K-iPSCs were induced and propagated as described previously [62]. Briefly, K-iPSCs were split into 6-well plates at a density of 10^5^ cells per well on day 0. On day 1, the culture medium was changed to PSC neural induction medium (neurobasal medium containing neural induction supplement, Gibco, MD). The medium was changed every 2 days. On day 6–7 of neural induction, primitive NSCs were dissociated with Accutase (Gibco, MD) and replated on Geltrex matrix-coated 6-well plates at 10^5^ cells per well in NSC expansion medium (50% neurobasal medium, 50% advanced DMEM/F12, and neural induction supplement, Gibco, MD). The NSC expansion medium was changed every other day until confluence was achieved.

For the NSCs differentiation experiments, we used K-NSCs in passages 4-5 according to the published protocol with several modifications [63,64]. Briefly, the rAAV2-infected K-NSCs cells were detached using Accutase followed by plating on poly-l-Lysine (Gibco, MD) and laminin (20 µg/mL, Gibco, MD)-coated 6-well plates (5 × 10^5^ cells per well) in Neurobasal medium with CultureOne supplement (Gibco, MD), B27 supplement (Gibco, MD), GlutaMax (Gibco, MD), and 200 mM ascorbic acid (Sigma-Aldrich, St. Louis, MO, USA). From day 0 to the end of the differentiation (at least day 14), the differentiation medium was changed every other day.

Mycoplasma contamination was measured using MycAway Plus-Color One-Step Mycoplasma Detection Kit (Yeason, Shanghai, China) for all cell cultures periodically. There are no contaminated cells applied in the study.

### 4.3. Immunofluorescence Staining

Immunofluorescence staining was performed as described previously [65]. The K-NSCs were seeded on coverslips coated with Geltrex matrix in 6-well plates and grown to 80% confluency. The cells were fixed with 4% formaldehyde for 15 min at room temperature, and the coverslips were washed three times in phosphate-buffered saline (PBS). Then, the cells were permeabilized with 0.5% Triton-X-100 in PBS for 15 min and blocked with 1% bovine serum albumin (BSA) for 30 min. The cells were incubated at 4 °C overnight in 1% BSA containing the primary antibodies. The following day, the cells were washed three times in PBS followed by incubated in secondary antibody for one hours at room temperature (goat anti rabbit-488, goat anti rabbit-594, and goat anti mouse-488 (Gibco, MD, 1:500)). After being washed three times in PBS and air-dried, the coverslips were treated with Fluoroshield mounting medium with DAPI (Sigma-Aldrich, St. Louis, MO, USA). Fluorescence was detected using fluorescence microscopy (IRX60, Sunny Optical Technology, Yuyao, China) at appropriate wavelengths for 100ms and the intensity was analyzed with ImageJ software (V1.8.0).

### 4.4. Sanger Sequencing

The genomic DNA of K-NSCs was extracted using the EasyPure^®^ Genomic DNA Kit (Transgene, Beijing, China). The disease-related variants in GALC were analyzed by Sanger sequencing using primer A: CTCAGATATTGGATT, and primer B: ATCAGCTCATACCA, separately.

### 4.5. Flow Cytometry Assays

NSCs were dissociated into single cells by Accutase (Gibco, MD). The cells were washed and diluted in Dulbecco’s Phosphate-Buffered Saline (DPBS, Gibco, MD). Staining was carried out as described previously [66]. The antibodies used were the same as those used for immunofluorescence staining. The stained cells were analyzed on a C6 flow cytometer (BD). A total of 10,000 cells were counted, and all analyses were performed in triplicate. Data were analyzed with FlowJo software (V10).

### 4.6. rAAV Packaging and Purification

The plasmids (pAAV-Rep/Cap, pAAV-GFP, pHelper) were purchased from Addgene (Cambridge, MA, USA). The pAAV-Rep/Cap plasmid has the same Rep sequence and distinct Cap sequence, which encode capsid for different rAAV serotypes packaging. The nucleotide sequence of GALC was obtained from GenBank (National Center for Biotechnology Information, NCBI) and synthesized in Sangon Biotech (Shanghai, China), which was tagged with the His coding sequence in the 3′ terminal. The fragment was inserted into the pAAV plasmid using restriction enzyme EcoRI and XhoI to construct pAAV-GALC.

Recombinant AAV was produced as previously described [67]. Briefly, the total mass (24 µg) of three plasmids—pAAV-Rep/Cap plasmid (8 µg), pAAV-GFP or pAAV-GALC-His vector plasmid containing inverted terminal repeats (ITRs) (6 µg), and pHelper plasmid (10 µg)—were transfected into HEK293T cells with Neofect DNA transfection reagent (Neofect, Beijing, China). The virus was purified by iodixanol gradient centrifugation and dialyzed against PBS using centrifugation with column with a 100 kD cutoff (Millipore, MA, USA) [68,69,70].

### 4.7. rAAV Titer Measurement

Genomic titers of the rAAV serotype were determined by quantitative PCR (qPCR) as previously described [71,72]. TransStart Green qPCR SuperMix (Transgene, Beijing, China) was used. The primers applied for the ITR sequence were: forward: GGAACCCCTAGTGATGGAGTT; reverse: CGGCCTCAGTGAGCGA. The rAAV serotype was titrated according to the standard curve obtained from known concentrations of pAAV-GFP plasmid. A standard curve was considered reliable when the coefficient of determination (R^2^) was greater than 0.98.

### 4.8. Western Blotting

The purity of the rAAV serotype was examined by SDS-PAGE. rAAV samples with 1010 viral genomes were boiled in the 5 x oading buffer (100 mM Tris-HCl, pH 6.8, 4% SDS, 200 mM DTT, 0.2% bromophenol blue, and 20% glycerol) for 5 min, and loaded on a 10% polyacrylamide gel. The samples were separated by SDS-PAGE and analyzed via sequential Western blotting as described previously [70,73]. Briefly, the samples were transferred to the nitrocellulose membrane (Amersham Protran 0.45 µm Membrane; Cytiva, MA, USA). The membrane was blocked with 5% non-fat milk in Tris-buffered saline containing 0.05% Tween (TBST) and incubated with the primary antibody overnight. The membrane was washed thrice in TBST and incubated with the secondary horseradish peroxidase (HRP)-conjugated antibody (Santa Cruz, 1:3000) for one hour. Immunoreactive proteins were further detected by the ECL Western blotting substrate (Gibco, MD).

### 4.9. NSC Infection

K-NSCs were infected at a multiplicity of infection (MOI) of 10^5^ with recombinant AAV2 serotypes encoding GFP. Seven days post-infection, fluorescence of cells was detected using fluorescence microscopy (IRX60, Sunny Optical Technology) at appropriate wavelengths and the intensity was quantified with ImageJ software (V1.8.0). K-NSCs were infected with rAAV2 serotypes encoding GALC tagged with the 6xHis peptide at an MOI of 10^5^. The cell pellets were harvested 48 h post-infection and homogenized in 400 µL PBS with a Potter–Elvehjem homogenizer. GALC expression was analyzed via Western blotting.

### 4.10. GALC Enzymatic Assay

GALC enzyme assay was performed according to reported protocols [34]. Briefly, the reaction mixture contained 5 µg extracted protein with 75 µmol/L 4-MU β-d-galactopyranoside substrates (Aladdin, Shanghai, China) resuspended in 100 µL buffer A (0.1/0.2 mol/L citrate/phosphate buffer, pH 4.0). The reaction was incubated for 1 h at 37 °C and stopped by the addition of 150 µL buffer B (0.2 mol/L glycine/NaOH, pH 10.6). The fluorescence value was measured by a spectrofluorometer (λex 360 nm, λem 450 nm, Tecan). One enzyme unit was defined as 1 µmol/h of substrate hydrolyzed at 37 °C.

### 4.11. Determination of the Psychosine Concentration

The psychosine concentration of cells was assessed by Gene Denovo Biotechnology Co. (Guangzhou, China) as previously described with minor adaptations [74]. Briefly, 50 mg cell samples were placed in a 1.5 mL centrifuge tube, followed by grinding the sample at 60 Hz and adding 80% methanol solution. After vortex mixing, ultrasonic vibration, and standing at −40 °C, the samples were centrifugated at 12,000 rpm for 10 min. The supernatants were passed through a 0.22 μm filter and measured by liquid chromatography-tandem mass spectrometry (LC-MS/MS). The intensity was calculated using MultiQuant software (V3.0.3). A psychosine sample (Santa Cruz, CA, USA) was used to generate the standard curve.

### 4.12. Statistical Analysis

The results were analyzed with GraphPad Prism software (V9). A two-tailed Student’s *t* test was used to compare two groups. All data are presented as the means ± SDs from at least three independent experiments. ***, significant differences at *p* < 0.001.

## 5. Conclusions

In this study, we generated a novel human-derived in vitro neural cell model of Krabbe disease by inducing patient-derived iPSCs to K-NSCs. Moreover, the rAAV2 serotype was found to efficiently transduce K-NSCs and restore abnormal GALC enzymatic activity. Our results suggest that the potential application of rAAV2-mediated gene therapy in Krabbe disease warrants further investigation.

## Data Availability

The authors confirm that all data underlying the findings are fully available. Informed consent was obtained from all subjects involved in the study. Written informed consent has been obtained from the patient(s) to publish this paper.

## Supplementary Materials

The following supporting information can be downloaded at: https://www.mdpi.com/article/10.3390/ph16040624/s1, Figure S1: AAV titer and purity measurement. a, Workflow of rAAV production and purification followed by K-NSC infection. b, Titers of rAAV vectors were measured via qPCR and quantified with GraphPad Prism software (V9). Data are expressed as the mean ± SD (n = 5). c, Western blot analysis of purified AAV with antibodies against the C-termini of the rAAV capsid proteins VP1, VP2, and VP3; Figure S2: Psychosine concentrations were determined by LC‒MS/MS. a, Standard curve of the psychosine sample. b, The intensity of psychosine in infected K-NSCs was calculated with MultiQuant software (V3.0.3); Figure S3: Western blot analysis of the progeny of rAAV2-infected K-NSCs with antibodies against His (tagged with GALC) and Actin.
