# Supplementary material for: rAAV2-Mediated Restoration of GALC in Neural Stem Cells from Krabbe Patient-Derived iPSCs
Source: Pharmaceuticals (Basel). 2023 Apr 20;16(4):624. doi: 10.3390/ph16040624 (PMC10143348; doi:10.3390/ph16040624)
Supplement: Supplementary file 1 [file pharmaceuticals-16-00624-s001.zip › pharmaceuticals-2227025-supplementary.pdf]

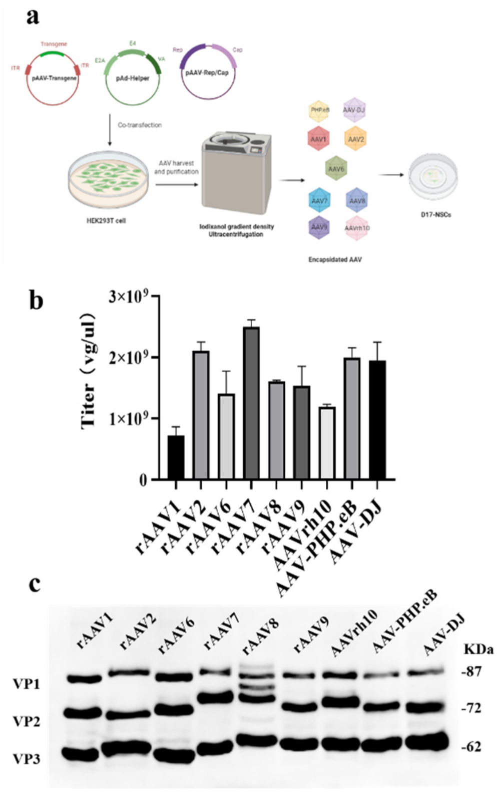

Supplementary Figure S1. AAV titer and purity measurement. **a**, Workflow of rAAV production and purification followed by K-NSC infection. **b**, Titers of rAAV vectors were measured via qPCR and quantified with GraphPad Prism software (V9). Data are expressed as the mean ± SD (n=5). **c**, Western blot analysis of purified AAV with antibodies against the C-termini of the rAAV capsid proteins VP1, VP2, and VP3.

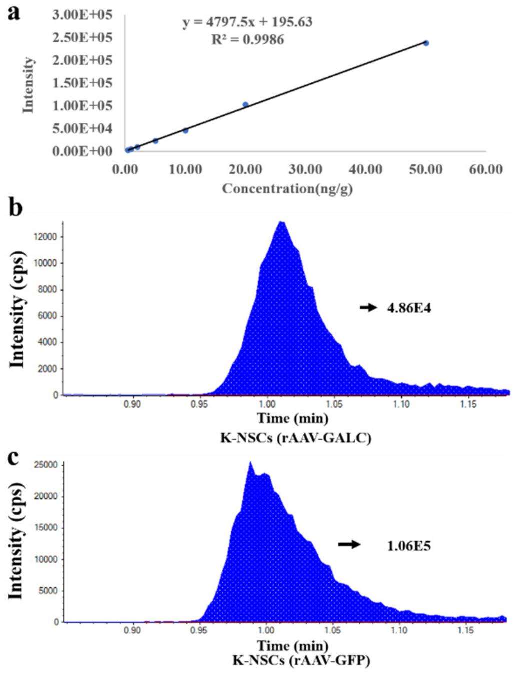

Supplementary Figure S2. Psychosine concentrations were determined by LC–MS/MS. **a**, Standard curve of the psychosine sample. **b**, The intensity of psychosine in infected K-NSCs was calculated with MultiQuant software (V3.0.3).

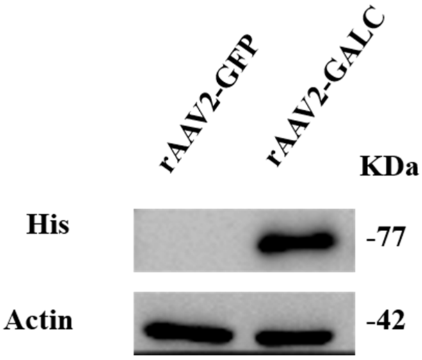

Supplementary Figure S3. Western blot analysis of the progeny of rAAV2-infected K-NSCs with antibodies against His (tagged with GALC) and Actin.
